# Engineering of Thermostable β‐Hydroxyacid Dehydrogenase for the Asymmetric Reduction of Imines

**DOI:** 10.1002/cbic.202000526

**Published:** 2020-09-16

**Authors:** Peter Stockinger, Luca Schelle, Benedikt Schober, Patrick C. F. Buchholz, Jürgen Pleiss, Bettina M. Nestl

**Affiliations:** ^1^ Institute of Biochemistry and Technical Biochemistry Department of Technical Biochemistry University of Stuttgart Allmandring 31 70569 Stuttgart Germany

**Keywords:** beta-HAD, imine reductases, promiscuity, substrate specificity, thermostability

## Abstract

The β‐hydroxyacid dehydrogenase from *Thermocrinus albus* (*Ta*‐βHAD), which catalyzes the NADP^+^‐dependent oxidation of β‐hydroxyacids, was engineered to accept imines as substrates. The catalytic activity of the proton‐donor variant K189D was further increased by the introduction of two nonpolar flanking residues (N192 L, N193 L). Engineering the putative alternative proton donor (D258S) and the gate‐keeping residue (F250 A) led to a switched substrate specificity as compared to the single and triple variants. The two most active *Ta*‐βHAD variants were applied to biocatalytic asymmetric reductions of imines at elevated temperatures and enabled enhanced product formation at a reaction temperature of 50 °C.

## Graphical abstract

**Get more from your enzyme**: A β‐hydroxyacid dehydrogenase from *Thermocrinus albus* was engineered to accept two different imine compounds as substrates. The variants were applied to biocatalytic asymmetric reductions at elevated temperatures leading to an enhanced product formation.

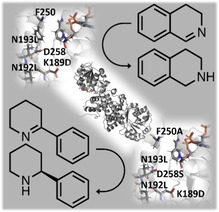

The asymmetric reduction of imines to allow the formation of chiral primary, secondary, and tertiary amines has been identified as a key area in synthetic organic chemistry.^[1]^ In this context, the development of enzymatic strategies is highly attractive because of their mild reaction conditions and their excellent selectivity (chemo‐, regio‐ and stereoselectivity). Successful biocatalysts have been reported for the synthesis of chiral amines from different enzyme classes, including lipases, monoamine oxidases, ω‐transaminases, amine dehydrogenases, ammonia lyases, engineered cytochrome P411, and imine reductases.^[2*–*8]^ In particular, imine reductases (IREDs) have emerged as a valuable new set of biocatalysts for the asymmetric synthesis of optically active amines.[^1^, ^9^, ^10^, ^11^, ^12^, ^13^, ^14^, ^15^, ^16^, ^17^] IREDs and related reductive aminases have been applied in biotransformations and enzyme cascades for the synthesis of chiral amines.[^18^, ^19^, ^20^, ^21^, ^22^, ^23^, ^24^, ^25^, ^26^, ^27^, ^28^, ^29^]

The use of sequence‐based bioinformatics classification approaches enabled the identification of characteristic sequence motifs and the annotation of putative IREDs in the Imine Reductase Engineering Database (https://ired.biocatnet.de/*)*.[^30^, ^31^] A deeper analysis of the sequence space of IRED homologues revealed a significant sequence similarity and a similar quaternary structure of β‐hydroxyacid dehydrogenases (βHADs).^[32]^ In addition to IREDs, several short‐chain dehydrogenases (SDRs) were reported to catalyze imine reduction.[^33^, ^34^] In contrast to βHADs, SDRs lack the sequence and structural similarities to IREDs and βHADs and constitute a separate, large enzyme family (https://sdred.biocatnet.de/).^[35]^ By systematically comparing imine‐reducing representatives of IREDs, βHADs, and SDRs, common principles were derived to support the targeted engineering of SDRs and βHADs into imine‐reducing enzymes (Scheme 1).^[36]^ The existence of alternative proton donors in IREDs and βHADs was proposed, and functional‐relevant residues were identified. These flank the active site and mediate the local electrostatic fine‐tuning of the catalytic residues.^[36]^ Exchanging several flanking residues in a dehydrogenase with distinct carbonyl‐reducing activity resulted in a promiscuous enzyme with imine‐reducing activity.^[37]^ Recently, three new imine‐reducing enzymes were generated by introducing single‐point mutations into βHADs, including the exchange of the proton donor and the removal of a bulky gate‐keeping residue.^[38]^ To access imine reduction in the glyoxylate reductase from *Arabidopsis thaliana* (*At*‐βHAD), the catalytically essential lysine at position 170 (K170) was exchanged by aspartic acid.

**Scheme 1:**
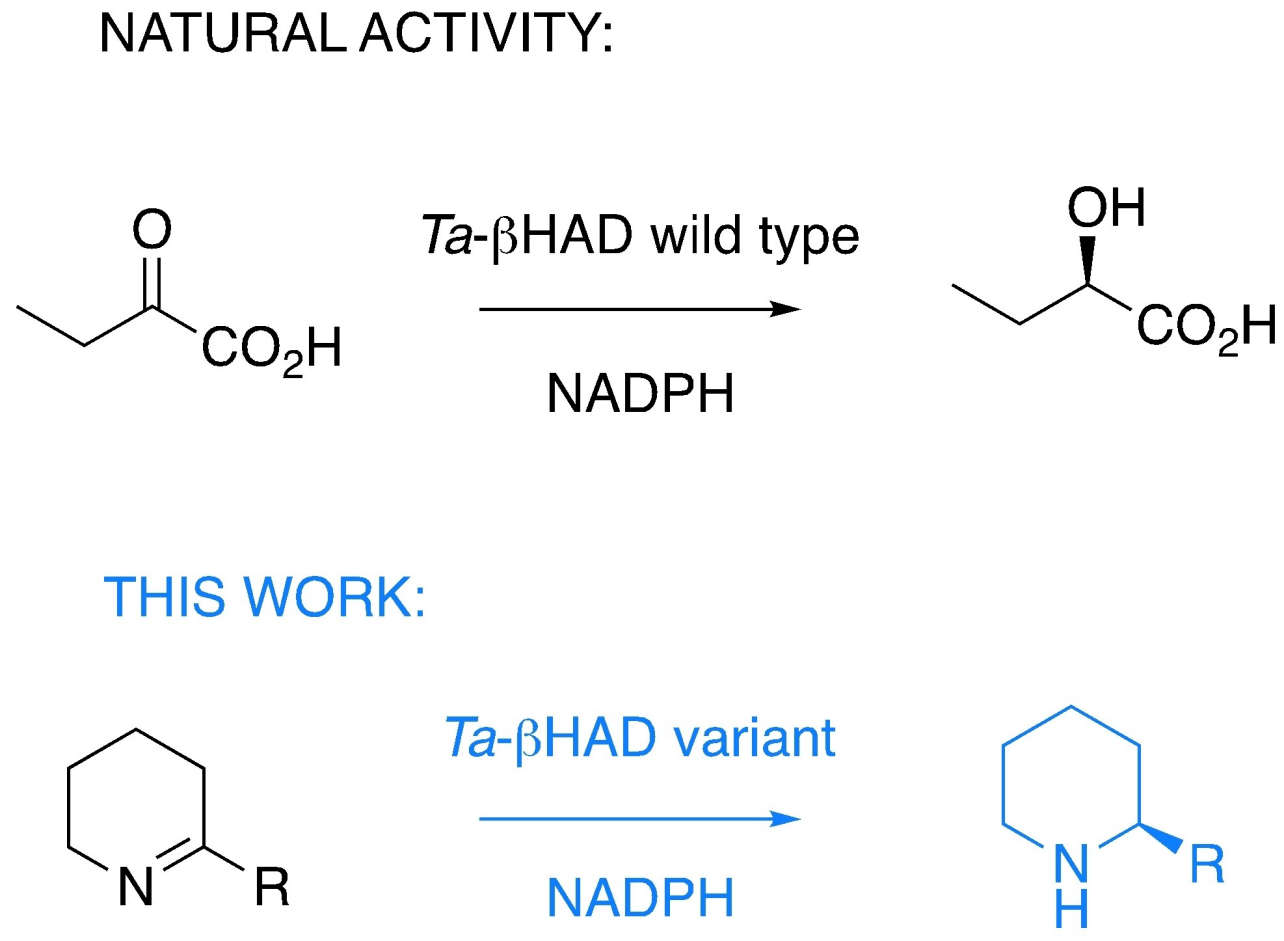
Natural asymmetric reduction of ketoacids by β‐hydroxyacid dehydrogenases and novel imine‐reducing activity by rational enzyme engineering.

Notably, imine reduction was also observed upon mutation of K170 to phenylalanine. The presence of the polar residues asparagine (N174) and aspartic acid (D240) might rationalize the observed imine‐reducing activity. This initial report established a proof‐of‐concept and highlighted limitations, notably low stability and expression level of the generated βHAD variants. *At*‐βHAD double‐variant K170D/N174L resulted in an unstable enzyme. Given the lack of polar interactions with N174, unfavorable repulsive energies between the neighboring aspartic acid residues are conceivable. Indeed, the destabilizing effect of the double variant K170D/N174 was compensated in the triple variant K170D/N174L/D239A and resulted in a catalytically active protein (https://doi.org/10.18419/opus‐10763).

We thus envisaged developing thermostable imine‐reducing enzymes for further application in the synthesis of chiral amines. As IREDs from thermophilic organisms are rare, the larger family of βHADs served as a starting point for thermostable‐enzyme candidates. Several βHAD homologues from hyperthermophiles were identified by comparison with the BacDive database.^[39]^ Four candidates were selected for further analysis (details of the sequence selection are given in the Supporting Information): *Tt*‐βHAD‐1 and *Tt*‐βHAD‐2 from *Thermus thermophilus*, *Ta*‐βHAD from *Thermocrinis albus* and *Pc*‐βHAD from *Pyrobaculum calidifontis* with pairwise protein sequence identities ranging from 27 to 36 % (see Tables S1 and S2 in the Supporting Information). As described previously,^[1]^, the four selected genes were synthesized carrying the lysine to aspartic acid mutation and subcloned into a pBAD‐33 vector followed by overexpression in *Escherichia coli* JW5510. After heat‐shock purification, the thermofluor assay^[40]^ with Sypro Orange was used to determine the thermostability. The thermal denaturation temperature of selected βHADs was about 60 °C (Table 1, Figures S6–S9).

**Table 1:** Thermostabilities (Tappm ) of variants.

Variant	Apparent melting temperature (Tappm )
*Tt*‐βHAD‐1K185D	61.8±0.2 °C
*Tt*‐βHAD‐2K187D	56.9±0.8 °C
*Ta*‐βHAD K189D	60.5±0.4 °C
*Pc*‐βHAD K190D	59.8±0.8 °C

Further, the oligomerization state was analyzed by size‐exclusion chromatography. While the *Pc*‐βHAD K190D variant was observed to form a dimer, *Tt*‐βHAD‐1, *Tt*‐βHAD‐2, and *Ta*‐βHAD variants were found to oligomerize as tetramers (Figures S2‐S5). To determine whether these single βHAD variants possessed activity towards imines, substrates 2‐methylpyrroline (**1**) and 3,4‐dihydroisochinoline (**2 a**) were first examined. None of the variants showed activity towards substrate **1**. Furthermore, we also evaluated the reduction of **1** using wild‐type enzymes. No promiscuous imine reduction activity was found. However, good product formation (60.5±1.0 %) was observed with imine **2 a** and variant *Ta*‐βHAD K189D at 25 °C and 24 h biotransformation time.

Engineering the active site of βHADs was effective to alter the catalytic activity and substrate specificity by using structural information to elucidate beneficial mutations and using multiple sequence alignments of enzyme homologues. In addition to single mutation K189D in *Ta*‐βHAD affording activity towards **3**, further positions were considered to play an important role in catalytic activity and even altering substrate specificity.^[38]^ Given the previous observation that the K170D/N174L/D239A mutation in *At*‐βHAD rendered the instable K140D/N174L variants active (https://doi.org/10.18419/opus‐10763), residues N192 and N193 in *Ta*‐βHAD were selected as positions to introduce potentially beneficial nonpolar flanking residues. Moreover, residues D258 and F250, considered as a putative alternative proton donor and gate‐keeping residue, respectively, were included to perform combinatorial single‐site mutagenesis (Table S5). Figure 1 highlights residues (i. e., N192, N193, F250, and D258) that are proposed to be important for imine reduction in *Ta*‐βHAD.

**Figure 1:**
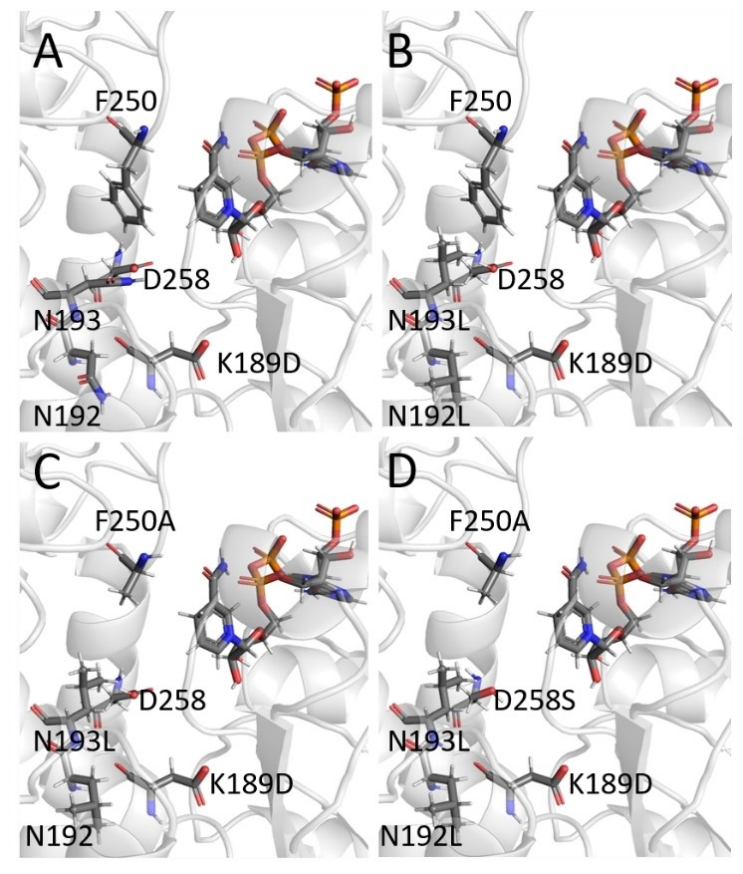
Imine‐reducing active‐site variants resulting from combinatorial mutagenesis of *Ta*‐βHAD visualized in a homology model. A) Single variant TA1 (K189D). B) Triple variant TA6 (K189D/N192L/N193L). C) Quadruple variant TA15 (K189D/N192L/N193L/F250A). D) Quintuple variant TA20 (K189D/N192L/N193L/F250A/D258S).

Considering these positions, an intuitively, focused combinatorial library was designed to further minimize the screening effort. In total, 20 variants were generated using *Ta*‐βHAD K189D as a template. These engineered and purified variants of *Ta*‐βHAD were tested with a panel of five imine substrates (Figure 2, Tables 1 and S8).

**Figure 2:**
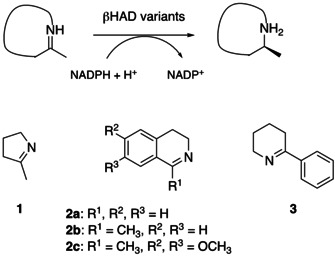
Imine substrates used in the asymmetric reduction with newly engineered *Ta*‐βHAD.

Biotransformations were analyzed by achiral and chiral GC (substrates **1** and **3**) and HPLC (substrates **2 a**–**2 c**). No activity was observed with substrate **1** as well as dihydroisoquinoline substrates **2 b** and **2 c**, possibly as a result of more sterically demanding methyl and methoxy substituents. Interestingly, two variants (TA4 and TA6) were found to exhibit improved activity for substrates **2 a** and **3**. For example, triple variant TA6 (*Ta*‐βHAD K189D/N192 L/N193L) showed high activity (>99 % product formation) with **2 a** after 4 h of biotransformation (Table 2, Figure S10). Remarkably, variant TA15 (*Ta*‐βHAD K189D/N192L/N193L/F250A) displayed activity on both substrates **2 a** and **3**. Improved conversions were obtained for **3** with various variants, whereas quintuple variant TA20 (*Ta*‐βHAD K189D/N192L/N193L/F25A/D258S) demonstrated the highest product formation. Product formation of 77 % and a high enantiomeric excess 99 % for the *S*‐product was detected after 24 hours of biotransformation (Figures S11 and S12).

**Table 2:** Activities of *Ta*‐βHAD variants towards 3,4‐dihydroisoquinoline (**2 a**) and 2‐phenylpiperideine (**3**) after 24 h reaction time.

Variant	Product formation [%] with		
	**1**, **2 b** and **2 c**	**2 a** ^[a]^	**3** ^[a]^
TA1	–	–	–
TA2	–	–	–
TA3	–	–	–
TA4	–	67.1±6.7	–
TA5	–	–	–
TA6	–	96.3±4.8	–
TA7	–	–	–
TA8	–	–	–
TA9	–	–	–
TA10	–	–	–
TA11	–	–	–
TA12	–	–	–
TA13	–	–	–
TA14	–	–	–
TA15	–	32.0±0.1	43.9±4.0
TA16	–	–	–
TA17	–	–	13.1±0.7
TA18	–	–	13.9±0.2
TA19	–	–	44.9±1.0
TA20	–	–	76.6±2.0

[a] Reactions (150 μL) performed in Tris ⋅ HCl buffer (50 mM, pH 8) with substrates (5 mM), NADPH (2.5 mM), glucose‐6‐phosphate (25 mM), glucose‐6‐phosphate dehydrogenase (1 U mL^−1^), MgCl_2_ (2.5 mM), and *Ta*‐βHAD variants (5 mg mL^−1^). ^**b**^ Product formations were determined by HPLC and GC analysis; see the Supporting Information. n.a.: not applicable. −: no activity

The increased product formation for variant T4 supports the need for a nonpolar donor‐flanking residue. Variant T6 (Figure 1B) displays an additively increased product formation, which could also be rationalized by the need for a nonpolar environment of the proton donor, but also by an advantageous influence on the binding of the nonpolar substrates. Variant TA15 was the only variant that displayed productive conversions of both substrate **2 a** and **3**. As the only difference compared to variant T6 was introduced by the additional mutation F250A, an erosion of steric effects negatively influencing the binding of substrate **3** is assumed. The drop of activity observed for substrate **2 a** might be rationalized by the direct involvement of F250 in productive binding. Therefore, the term gate‐keeper does only apply for the imine‐reducing activity toward substrate **3**. Interestingly, the product formation with substrate **2 a** is lost with the additional mutations D258A or S in variants T17–T20, which supports the hypothesis of alternative proton donors in βHADs.^[1]^ However, it cannot be excluded that the altered steric and electrostatic properties of this additional mutation hinder the productive binding of substrate **2 a** .

Finally, to demonstrate the thermostable imine‐reducing applicability of variants generated, the asymmetric reductions of imines **2 a** and **3** were performed at elevated temperatures. The reactions were compared for temperatures 25 and 50 °C using variants TA6 and TA20 in the reductions of substrates **2 a** and **3**, respectively. As a full conversion of **2 a** was observed with TA6 at 25 °C within 4 h reaction time, the amount of enzyme was reduced to monitor the impact of temperature on enzyme activity. Thus, the enzyme concentrations were lowered (TA6 1 mg mL^−1^ and TA20 2.5 mg mL^−1^) to avoid full product formation (Table 3). Improved asymmetric reduction of **2 a** and **3** at 50 °C was achieved with 50 and 30 % product formations, respectively. These results show an increase of 27 % product formation with TA4 (imine **2 a**) and 85 % with TA 20 (imine **3**), while selectivity remained unchanged (99 % *ee* for *S*‐amine product). To monitor the initial reduction activities towards imines **2 a** and **3** at elevated temperatures, lower enzyme concentrations were used.

**Table 3:** Comparison of reductions of imines **2 a** and **3** at elevated temperatures using the two best variants after 4 h reaction times.

	Variant	Substrate^[a]^	*T* [°C]	Amine product [%]^[b]^	*ee* [%]^[b]^
1	TA6	**2 a**	25	38.7±0.6	n.a.
2	TA6	**2 a**	55	49.5±2.8	n.a.
3	TA20	**3**	25	16.1±1.2	99 (*S*)
4	TA20	**3**	55	29.6±1.4	99 (*S*)

[a] Reactions (150 μL) performed in Tris ⋅ HCl buffer (50 mM, pH 8) with substrates (5 mM), NADPH (2.5 mM), glucose‐6‐phosphate (25 mM), glucose‐6‐phosphate dehydrogenase (1 U mL^−1^), MgCl_2_ (2.5 mM), and *Ta*‐βHAD variants TA6 (1 mg mL^−1^) and TA20 (2.5 mg mL^−1^). [b] Product formations and *ee* values were determined by HPLC and GC analysis; see the Supporting Information. n.a.: not applicable

In summary, we were able to generate further imine‐reducing variants from the βHAD family. Utilizing the biological diversity of this large enzyme family enabled the identification of a stable template, which revealed to be highly substrate‐specific. Utilizing previously gained insights into the local electrostatic pattern occurring in the substrate‐binding site of imine‐reducing enzymes enabled to minimize the screening effort and resulted in a switch of substrate specificity. Thereby, an excellent enantiomeric excess (>99 %) of product *S*‐2‐phenylpiperidine was provided. To the best of our knowledge, this exceeds the selectivities of the yet described IREDs toward the respective substrate. We expect that the reported basic activities can be further improved by computational design or random mutagenesis to complement the desired stability properties with reasonable conversions.

## Conflict of interest

The authors declare no conflict of interest.

## Supporting information

As a service to our authors and readers, this journal provides supporting information supplied by the authors. Such materials are peer reviewed and may be re‐organized for online delivery, but are not copy‐edited or typeset. Technical support issues arising from supporting information (other than missing files) should be addressed to the authors.

Supplementary
