# Supplementary material for: Engineering of Thermostable β‐Hydroxyacid Dehydrogenase for the Asymmetric Reduction of Imines
Source: Chembiochem. 2020 Sep 16;21(24):3511–4. doi: 10.1002/cbic.202000526 (PMC7756219; doi:10.1002/cbic.202000526)
Supplement: Supplementary file 1 — Supplementary [file CBIC-21-3511-s001.pdf]

ChemBioChem

Supporting Information

Engineering of Thermostable β-Hydroxyacid Dehydrogenase for the Asymmetric Reduction of Imines

Peter Stockinger[+], Luca Schelle[+], Benedikt Schober, Patrick C. F. Buchholz, Jürgen Pleiss, and Bettina M. Nestl*

Supporting Information

Content

I. SEQUENCE SELECTION AND PLASMIDS 2

II. GENERATION OF *TA*-BHAD VARIANTS..... 6

III. PROTEIN EXPRESSION AND PURIFICATION..... 8

IV. THERMOFLUOR ASSAY..... 9

V. SIZE-EXCLUSION CHROMATOGRAPHY 9

VI. THERMOFLUOR ASSAY..... 12

VII. BIOTRANSFORMATIONS 15

BIOTRANSFORMATIONS IN CO-SOLVENTS..... 15

VIII. MOLECULAR MODELING..... 19

IX. REFERENCES 21

I. Sequence selection and plasmids

A set of homologous β-hydroxyacid dehydrogenases (βHAD) sequences from Fademrecht et al.[1] was searched for sequences originating from hyperthermophilic archaea or bacteria by comparison with the BacDive database (release 09/2017).[2] The hypothesis was that sequences from hyperthermophiles would likely encode for thermostable proteins. Archaeal sequences were only considered if corresponding crystal structures were available in the Protein Data Bank (PDB) to increase the chances for protein expression in *E. coli*.

Based on the previously established standard numbering scheme for imine reductases,[1] equivalent position numbers can be assigned with the imine reductase from *Streptomyces kanamyceticus* (PDB accession 3ZHB) as a reference sequence. Only sequences containing a lysine residue at standard position 187 and a glutamine or asparagine residue at standard position 191 were considered for further analysis (Table S1). Both positions were described as relevant for substrate interaction in βHADs in previous works.[3–5]

Table S1: Plasmids used for heterologous expression of the βHAD variants.

Plasmid	βHAD variant	Source organism	NCBI accession
pET28a_*Tt*-βHAD-1_K185D	*Tt*-βHAD-1 K185D	*Thermus thermophilus*	WP_143584305
pET28a_*Tt*-βHAD-2_K187D	*Tt*-βHAD-2 K187D	*Thermus thermophilus* HB8	WP_011174107
pET28a_ *Ta*-βHAD_K189D	*Ta*-βHAD K189D	*Thermocrinis albus* DSM 14484	WP_012992122
pET28a_ Pc-βHAD_K190D	*Pc*-βHAD K190D	*Pyrobaculum calidifontis* JCM 11548	3WS7_A

Four candidates for thermostability were selected for experimental validation: Two candidates from *Thermus thermophilus* (Tt-βHAD-1 and Tt-βHAD-2), one candidate from *Thermocrinis albus* (Ta-βHAD), and an archaeal candidate with known crystal structure from *Pyrobaculum calidifontis* (Pc-βHAD, with PDB accession 3WS7). The four selected βHAD candidates were found to share modest sequence identities between 26.5 and 35.7 % (Table S2). The wild-type sequences of the two candidates Pc-βHAD and Tt-βHAD-1, were found to also contain a phenylalanine residue at standard position 250, which was described as 'gate-keeper' for the access of bulkier substrates in previous works.[3]

Table S2: Pairwise global sequence identities for the wild-type sequences of the four βHAD variants under investigation (Table S1). Pairwise Needleman-Wunsch alignments were performed using the implementation of the software suite EMBOSS (version 6.6.0)[6] with gap opening and extension penalties of 10 and 0.5, respectively, and the BLOSUM62 substitution matrix.

	Ta-βHAD	*Tt*-βHAD-1	*Tt*-βHAD-2
Pc-βHAD	35.7	26.5	32.6
Ta-βHAD		26.9	27.9
Tt-βHAD-1			33.1

Selected enzymes were synthesized from BioCat GmbH (Germany) containing the replacement of the catalytic lysine by an aspartic acid (highlighted in green in the following sequence data). Codon-optimized synthetic genes were cloned into a pET-28 vector system.

Tt-βHAD-1

DNA sequence:

ATGGGTAGCAGCCATCATCATCATCACCATAGCAGTGGCCTGGAAGTTCTGTTTCAGGG
CCCGGCAATGGGTCGCATTGGTTTTATTGGCCTGGGCCGCATGGGTCTGCCGATGGCT
CAGAATCTGGCAAAAGCAGGTTATGAAGTGCTGGCATGGAATCGTACCCCGAAAGAAG
TGGAAGGCCTGACCCTGGTGGGTAGCCCGCGCGAAGCCGCAGGTACCGGTCTGGTTA
TTACCATGCTGGCAGATGATGCAGCAACCGAAGCCGTTCTGGGCGAAATTCTGGAAGG
TCTGCCGGAAGGCGGTCTGCATATTGCAATGAGCACCCTGGGCGTTCCGTATAGCCGC
GCCCTGGAAGAACGCCATAAAGCCGCCGGTCGTCGCTATCTGGCAGCACCGGTGTTTG
GCCGCCCGGAAGCAGCAGCAAGTCGCGCTCTGCGTATTGTTGTGGCCGGCGAAGCAC
GCGATGTGGAAGAAGCCCGTCCGATTCTGAGTAGCCTGGGTCAGGAAGTTCATGTTGT
TGGTGAACGCCCGCATCAGGCACATGCAGTTGATCTGGGTGGTAATTTTCTGATTGCAG
GTATGCTGGAAGCCCTGAGTGAAGCATATGTTCTGGTGGAAAAGAATGGCGTGAAACG
CGAAGCCTTTTATGAAGTGGTGCGTGCATTTTTCCGCAGCCCGGTGTATGAAAGTTATG
GTCGCATTCTGCTGGAACGCCGCTTTACCCCGCCGGGCGCTGCTTTACGTCTGGGCCT
GAAAGATGTGCGCCTGATTCATCAGGCCGCAGATACCAGTCATACCCCGATGCCGCTG
GCACATCTGCTGCTGGATCGTATGCTGGAAGGCGTGGCCCGTGGCATGGGCGAAGAA
GATTGGGCAGCCGTTCTGAAAGTGGTGGAAGGTAGCGCCGGTATTGGTGGCTAA

Protein sequence:

MGSSHHHHHHSSGLEVLFQGPAMGRIGFIGLGRMGLPMAQNLAKAGYEVLAWNRTPKEVE
GLTLVGSPREAAGTGLVITMLADDAATEAVLGEILEGLPEGGLHIAMSTLGVPYSRALEERHK
AAGRRYLAAPVFGRPEAAASRALRIVVAGEARDVEEARPILSSLGQEVHVVGERPHQAHAV
DLGGNFLIAGMLEALSEAYVLVEKNGVKREAFYEVVRAFFRSPVYESYGRILLERRFTPPGA
ALRLGLKDVRLIHQAADTSHTPMPLAHLLLDRMLEGVARGMGEEDWAAVLKVVEGSAGIGG

Tt-βHAD-2

DNA sequence:

ATGGGCAGCAGTCATCATCATCATCACCATAGTAGTGGTCTGGAAGTGCTGTTTCAGGG
TCCGGCCATGGAAAAAGTGGCATTCATTGGTCTGGGTGCAATGGGTTATCCGATGGCA
GGTCATCTGGCCCGTCGTTTTCCGACCCTGGTGTGGAATCGCACCTTTGAAAAAGCCCT
GCGCCATCAGGAAGAATTTGGTAGTGAAGCCGTGCCGCTGGAACGCGTGGCAGAAGC
ACGTGTTATTTTTACCTGCCTGCCGACCACCCGTGAAGTGTATGAAGTGGCAGAAGCGC
TGTATCCGTATCTGCGTGAAGGTACCTATTGGGTGGATGCCACCAGCGGTGAACCGGA
AGCAAGCCGCCGCCTGGCCGAACGTCTGCGTGAAAAAGGTGTGACCTATCTGGATGCA
CCGGTTAGTGGTGGCACCAGCGGCGCCGAAGCCGGTACACTGACCGTTATGCTGGGC
GGCCCGGAAGAAGCCGTTGAACGCGTTCGCCCGTTTCTGGCCTATGCAAAAAAGGTGG
TGCATGTGGGCCCGGTTGGTGCCGGTCATGCCGTTGATGCCATTAATAATGCCCTGCT
GGCAGTTAATCTGTGGGCAGCCGGCGAAGGTCTGCTGGCACTGGTGAAACAGGGCGT
TAGTGCCGAAAAAGCACTGGAAGTTATTAATGCAAGTAGCGGCCGCAGTAATGCAACCG
AAAATCTGATTCCGCAGCGCGTTCTGACCCGTGCATTTCCGAAAACCTTTGCCCTGGGT
CTGCTGGTGAAAGATCTGGGTATTGCCATGGGCGTGCTGGATGGTGAAAAAGCACCGA
GCCCGCTGCTGCGTCTGGCACGTGAAGTGTACGAAATGGCAAAACGTGAACTGGGTCC
GGATGCCGATCATGTTGAAGCACTGCGCCTGCTGGAACGCTGGGGTGGTGTTGAAATT
CGCTAA

Protein sequence:

MGSSHHHHHHSSGLEVLFQGPAMEKVAFIGLGAMGYPMAGHLARRFPTLVWNRTFEKALR
HQEEFGSEAVPLERVAEARVIFTCLPTTREVYEVAEALYPYLREGTYWVDATSGEPEASRR
LAERLREKGVTYLDAPVSGGTSGAEAGTLTVMLGGPEEAVERVRPFLAYAKKVVHVGPVG
AGHAVDAINNALLAVNLWAAGEGLLALVKQGVSAEKALEVINASSGRSNATENLIPQRVLTR
AFPKTFALGLLVKDLGIAMGVLDGEKAPSPLLRLAREVYEMAKRELGPDADHVEALRLLERW
GGVEIR

Ta-βHAD

DNA sequence:

ATGGGTAGCAGTCATCATCATCATCACCATAGCAGCGGTCTGGAAGTTCTGTTTCAGGG
TCCGGCCATGCGCGTTGGTTTTATTGGCCTGGGCAGCCTGGGCAAAACCATTGCACGT
CGTCTGCTGGATCAGGGCGTGCCGCTGATTGTTTGGAATCGCACCCGCGAAAAAGCCG
CAGATCTGGGCGTTCCGGTTGCCGAAAGTCCGGCCGATCTGATTAAGCAGGTGGATGT
GGTGCTGATGATTGTTTTTGATAGCGCAGCAAGCGAAGAAGTTATTCTGGGCAAAGGCG
GCCTGATTGAAGGTGGCGTTAAAGGTAAAGTGGTGGTTGATATGACCACCAATCATTTT
GCCTTTCCGCCGCTGGCCTATCGTGAAATTAAGGGCCGTGGTGGTTTTTATCTGGATGC
ACCGGTTCTGGGTAGCGTGGTGCCGGCCCAGCGTGGTGAACTGGTTATGCTGGTTGGT
GGTGACGAAGAAAAACTGCGTGAAGTGCGCCCGATTCTGGAACGCTTTTGTCGTAAAAT
CTATTATGTGGGCGAAGCAGGTAAAGCCACCCAGCTGGATCTGATTAATAATATTGTTCT
GGGCGGTATTATGGAAGTTCTGGCAGAAGCCATTGCCATTGCAGAAAAAGCAGGTTTTG
ATCGCCAGCTGGTTATTGATGTTCTGAATGATGGCGCAGGCAAAAGTTATATTCTGGAT
GTTAAACGCGAAAAACTGCTGCGCGAAGATTTTAGCACCCATTTTAGTGTGGATCTGATT
CATAAAGATCTGCATTATGCCCAGGATCTGATTAAGGAACTGGGCGTGTTTAGCTTTAG
CGTTCAGAATATTAAGGAAGCATATGGTTTTGCAAAGGCCATGGGTATGGGTAAAGAAG

ATTTTTCAGCAGTGCTGGGCGCCCTGCTGAGTGCCTATTTTTATAAAGGCGGTAAAGTG
TAA

Protein sequence:

MGSSHHHHHHSSGLEVLFQGPAMRVGFIGLGSLGKTIARRLLDQGVPLIVWNRTREKAADL
GVPVAESPADLIKQVDVVLMIVFDSAASEEVILGKGGLIEGGVKGKVVVDMTTNHFAFPPLAY
REIKGRGGFYLDAPVLGSVVPAQRGELVMLVGGDEEKLREVRPILERFCRKIYYVGEAGKAT
QLDLINNIVLGGIMEVLAEAIAIAEKAGFDRQLVIDVLNDGAGKSYILDVKREKLLREDFSTHFS
VDLIHKDLHYAQDLIKELGVFSFSVQNIKEAYGFAKAMGMGKEDFSAVLGALLSAYFYKGGK
V

Pc-βHAD

DNA sequence:

ATGGGTAGTAGTCATCATCATCATCACCATAGCAGTGGTCTGGTTCCGCGTGGCAGCCA
TATGCGCGTGGGCTTTATTGGCCTGGGCATTATGGGTGGCCCGATGGCAACCCATCTG
CTGAAAGCAGGCTTTCTGGCAGCAGTTTATAATCGTACCCGCGAAAAAACCAAACCGTT
TGCAGAAGCCGGTGTGTATGTGGCCGAAAGTCCGGCCGATCTGGCCAAACGTGTGGAT
GTGGTTATTGTGATGGTGAGCGATGCACCGGATGTTGAACAGGTTCTGTTTGGCCCGA
GTGGCGTGGTGGAAGGTGCACGTCCGGGCCTGATTGTTGTTGATATGAGCACCAATAG
CCCGGATTGGGCACGCAAATTTGCCGAACGCCTGGCACAGTATGGTATTGAATTTCTGG
ATGCACCGGTGACCGGTGGTCAGAAAGGTGCAATTGAAGGCACCCTGACCATTATGGT
TGGTGGCAAAGAAGAACTGTTTCATCGCCTGCTGCCGATTTTTAAAGCAATGGGCCGCG
ATATTGTTTATATGGGCCCGGTTGGTTATGGTCAGGCAATGGATCTGGTTAATCAGGTT
GTTGTTGCCCTGAATACCGTGGCAATGGTTGAAGGCCTGAAACTGGCAAAAGCCCTGG
GCCTGGATATGGATAAAGTTGCAGAAGTTCTGACCCGCGGCGCCGCACGCAGTGGTGC
AATTGAGCTGTATCTGCCGAAACTGCTGAAAGGTGACCTGAGCCCGGGCGCAAAAGCC
GAACATCTGAAAAAAGATCTGGGTTATGTGCTGGAAGAAGCCCGCAAACGCGGTGTGA
AACTGCCGGGCGCAGAACTGGCCTATGAACTGTATCGCAAAATGGTGGAAGATGGTGC
CGGCAGTCTGGGTATTCATGCCCTGGGTTTTTAT

Protein sequence:

MGSSHHHHHHSSGLVPRGSHMRVGFIGLGIMGGPMATHLLKAGFLAAVYNRTREKTKPFA
EAGVYVAESPADLAKRVDVVIVMVSDAPDVEQVLFGPSGVVEGARPGLIVVDMSTNSPDW
ARKFAERLAQYGIEFLDAPVTGGQKGAIEGTLTIMVGGKEELFHRLLPIFKAMGRDIVYMGPV
GYGQAMDLVNQVVVALNTVAMVEGLKLAKALGLDMDKVAEVLTRGAARSGAIELYLPKLLK
GDLSPGAKAEHLKKDLGYVLEEARKRGVKLPGAELAYELYRKMVEDGAGSLGIHALGFY

II. Generation of *Ta*-βHAD variants

pET28a_*Ta*-βHAD_K189D served as the starting template for the creation of several active site variants. Site-directed mutagenesis was performed using the QuikChange Site-Directed Mutagenesis method.[7] The used reaction mix and the thermocycler program (Mastercycler epgradient, Eppendorf) are listed in Tables S3 and S4, respectively.

Table S3: Reaction mix for site-directed mutagenesis

Components	50 µl Reaction	Final Concentration
10X PfuUltra II Reaction Buffer	5 µl	1x
10 mM dNTPs (each)	1.25 µl	250 µM
Template DNA	50-100 ng	
PfuUltra II Fusion HS DNA Polymerase	1 µl	0.05 U/µl
DMSO	1 µl	
10 µM Forward Primer	1.5 µl	0.5 µM
10 µM Forward Primer	1.5 µl	0.5 µM
Nuclease-free ddH$_2$O	ad 50 µl	0.5 µM

Table S4: Thermocycler program for site-directed mutagenesis

Temperature	Time [s]	Cycles
95	120	
95	30	30x
55*	30	30x
72	220**	30x
72	360	
8	hold	

*annealing temperature for all used primer pairs
**adjusted to 35 s/1 kb template

After the PCR 2 µl (2 U) of *Dpn*I was directly added to the reaction mix, and the methylated parental template DNA was digested for 1h at 37°C. The transformation was performed by the heat-shock of chemocompetent *E. coli* JW5510. The regenerated cells were fully plated on LB$_{Cmp}$ agar plates and incubated at 37 °C overnight. Overnight cultures of single clones were used for plasmid isolation with the Zyppy$_{TM}$ Plasmid Miniprep Kit (Zymo Research) according to the manufacturer's protocol. The validation of the introduced mutations into the plasmids was performed by Eurofins (Ebersberg, Germany) via Sanger sequencing with standard primer(s) pTRCHis-RP and pTRCHis-RP.

Table S5: Overview of generated and tested variants of *Ta*-βHAD

ID	Mutations
**TA1**	K189D
**TA2**	K189D/F250A
**TA3**	K189D/D258S
**TA4**	K189D/N192L
**TA5**	K189D/N193L
**TA6**	K189D/N192L/N193L
**TA7**	K189D/N193L/N192L/D258A
**TA8**	K189D/N193L/N192L/D258S
**TA9**	K189D/F250A/N192L
**TA10**	K189D/F250A/N193L
**TA11**	K189D/F250A/D258A
**TA12**	K189D/F250A/D258S
**TA13**	K189D/F250A/N192L
**TA14**	K189D/F250A/N193L
**TA15**	K189D/F250A/N192L/N193L
**TA16**	K189D/F250A/N192L/D258A
**TA17**	K189D/F250A/N193L/D258A
**TA18**	K189D/F250A/N192L/D258S
**TA19**	K189D/F250A/N193L/D258S
**TA20**	K189D/F250A/N193L/N192L/D258S

III. Protein expression and purification

Pre-cultures in 5 ml LB (34 µg/mL Cmp) were inoculated with 100 µL over-night cultures of sequenced single clones and incubated at 37 °C and 180 rpm for 20 h. The main culture, 800 ml TB (34 µg/ml Cmp) in 2 l flasks with baffles (VWR), was inoculated with 1 mL of pre-culture. The main culture was grown at 37 °C and 150 rpm until an OD of 0.7-0.8. The overexpression was induced by the addition of L-(+)-arabinose in a final concentration of 0.03 % (*w/v*) and incubated for 18 h at 25 °C and 150 rpm. After 18 h, the cells were placed on ice, and the cell suspension was centrifuged for 30 min at 10000 g (Avanti J-26S XP centrifuge, Beckmann Coulter). The supernatant was discarded, and the cells were washed with 25 ml KP_i-buffer (50 mM, pH 7.5). The washed cells were then transferred to 50 mL plastic tubes (Sarstedt) and centrifuged for 30 min at 3220 g (Centrifuge 5810 R, Eppendorf). The supernatant was discarded.
The cell pellets were resuspended in 2 ml of buffer TRIS-HCl (50 mM, pH 8) for *Ta*-βHAD variants per gram cell pellet. The resuspended cells were disrupted by high-pressure homogenizer (EmulsiFlex C5, Avestin) at 4 °C for 3 cycles with 750-1000 bar counter-pressure. The disrupted cells were centrifuged for 30 min at 4 °C and 8000 g (Centrifuge 5810 R, Eppendorf). The obtained lysates were further processed or stored at -20 °C.
Since *Ta*-βHAD variants are thermostable, they were purified using an optimized heat-treatment purification protocol. The finally obtained protocol is described below. The lysates of the *Ta*-βHAD variants were incubated at 57 °C for 60 min for precipitation of *E. coli* proteins. The sample with the precipitate was centrifuged for 30 min at 4 °C and 8000 g (Centrifuge 5810 R, Eppendorf) to get rid of all precipitated proteins. The supernatant was then transferred into Vivaspin 6 centrifugal concentrators (10.000 MWCO PES, Sartorius) and centrifuged at 6000 g (Centrifuge 5810 R, Eppendorf) until approximately 1 ml remained. Then 5 mL of TRIS-HCL buffer (50 mM, pH 8.0, and 10 % (*w/v*) L-sorbitol) was added two times and centrifuged until approximately 1 mL concentrated, and the re-buffered enzyme was obtained. For determination of the protein concentration of the βHAD variants, the Pierce™ BCA Protein Assay Kit (Thermo Scientific) was used according to the manufacturer's protocol. BSA was used as a calibrating agent with known protein concentrations. The soluble expression of the *Ta*-βHAD variants (34 kDa) was validated via SDS-PAGE gel analysis.

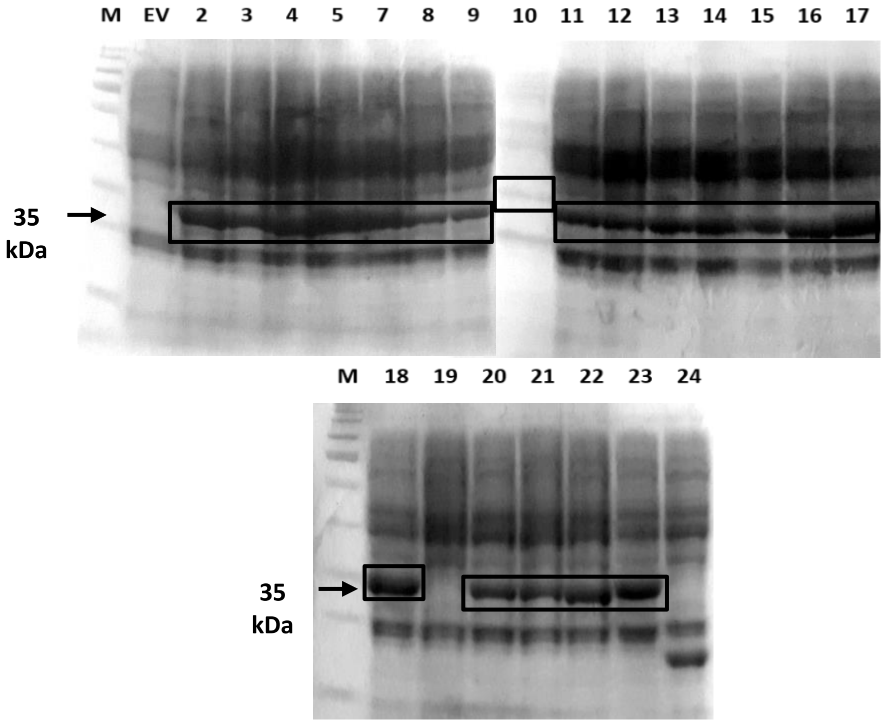

Figure S1: SDS-PAGE gel analysis of *Ta*-βHAD variant expression. The SDS-PAGE gel (12 %) of the *Ta*-βHAD variant lysates was stained with Coomassie blue. The expression was induced with 0.03% (*w/v*) L-arabinose for 20 h. The green boxes indicate the soluble expression of a variant. M: Protein marker.

IV. Thermofluor assay

The thermal shift (ThermoFluor$_{TM}$) assay offers a quick and simple technique for assessing the thermal stability of proteins. Sypro$_{TM}$ Orange was used as fluorescent dye to track protein unfolding with respect to temperature. Therefore 20 µL of purified enzyme variants and 5 µL of Sypro$_{TM}$ Orange dye were mixed in specific 96-well plates for real-time PCR (twin.tec PCR Plate 96, Eppendorf AG, Hamburg, Germany) to a final assay concentration of about 2 µM protein and 5x dye, respectively. The plates were immediately covered using optically clear adhesive sheets (Optical Adhesive Covers, Applied Biosystems, Foster City, US) and directly centrifuged at room temperature and 900 g for 2 min to collect solutions in the bottom of the well and to remove bubbles. The assay plate was analyzed using the Master cycler EP Gradient real-time PCR instrument (Eppendorf AG, Hamburg), setting following parameters: plate layout: well; filter 520 nm: SYBR; sample volume: 25 µL; PCR program: hold 20°C for 1 min, heat up with a heating rate of 2.4°C per minute to 99°C; total program time: 33 min. The instrument's software *realplex* determines the increase in fluorescence over time depending on the increase in temperature (melting curve), whereby the inflection point of the determined slope correlates with the melting temperature (T_M) of the protein.

V. Size-exclusion chromatography

Size exclusion chromatography for the analysis of the oligomerization state was performed on an Agilent 1260 Infinity II LC System using a Yarra 3µ SEC-200 column (300 x 4.6 mm, Phenomenex). The gel filtration standard #1511901 from Bio-Rad was used for calibration. The method is described in Table S6.

Table S6: HPLC method used for the analysis of the oligomerization state.

Mobile phase	flow rate in mL·min$_{-1}$	temperature
50 mM sodium phosphate buffer pH 6.5 containing 100 mM NaCl	0.3	40 °C

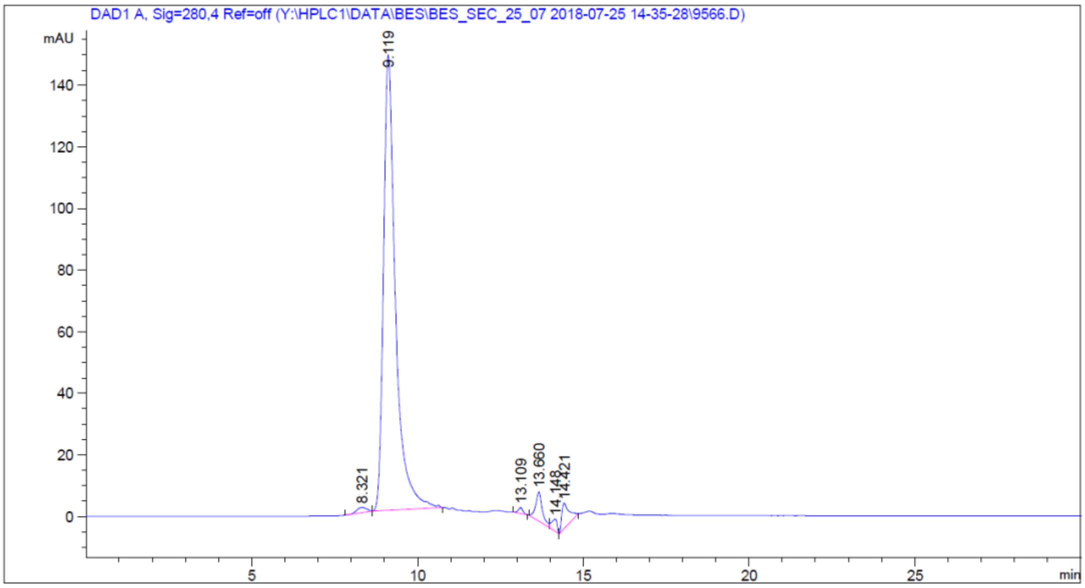

Figure S2: HPLC size-exclusion chromatography *of Tt*-βHAD-1 K185D.

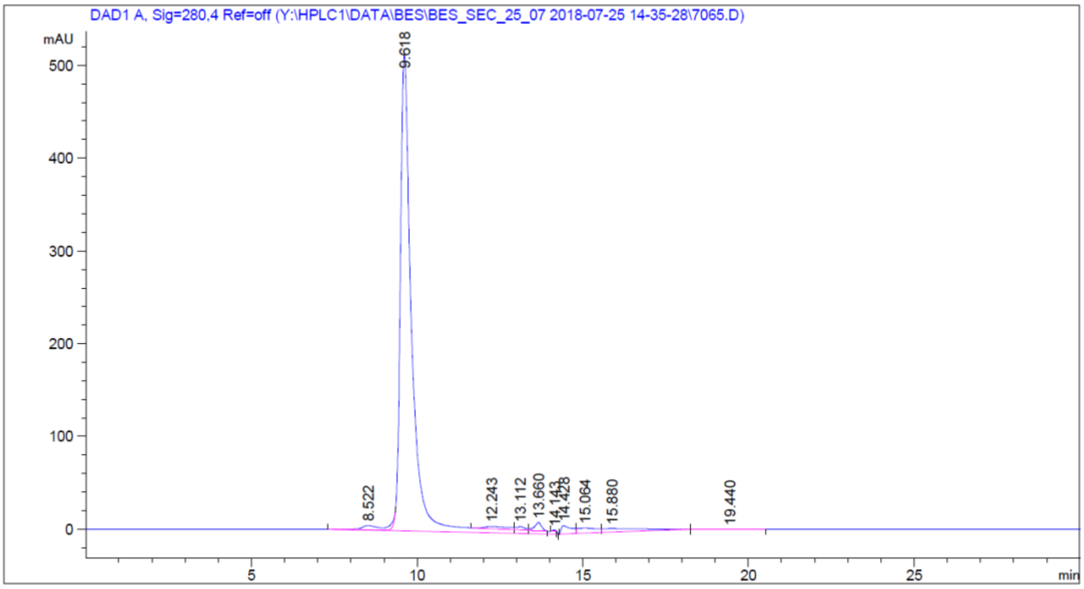

Figure S3: HPLC size-exclusion chromatography of *Tt*-βHAD-2 K187D.

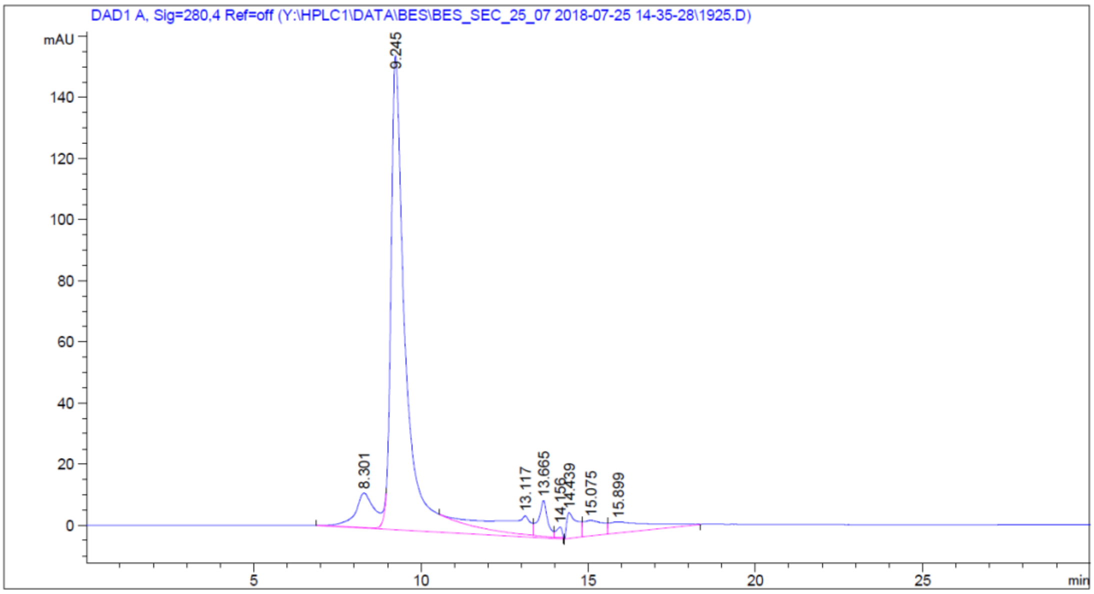

Figure S4: HPLC size exclusion chromatography of *Ta*-βHAD K190D.

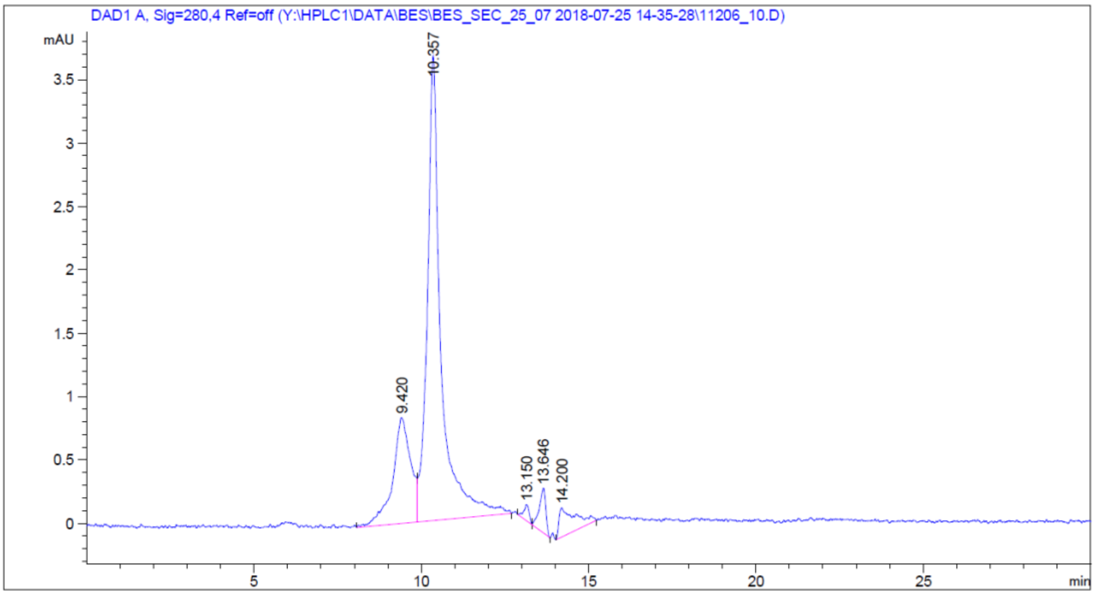

Figure S5: HPLC size exclusion chromatography of *Pc*-βHAD K190D.

VI. Thermofluor assay

Thermofluor assay for the analysis of the thermostability was performed using the realplex$_2$ Mastercycler (Eppendorf) and SYPRO Orange (Thermo Fisher Scientific). The purified enzyme was mixed with 2.5x SYPRO Orange and measured at 520 nm. The temperature program is described in Table S7.

Table S7: Temperature program of the thermofluor assay.

Heating rate	temperature	Hold time
-	20 °C	2 min
2.6 °C/min	99 °C	15 sec

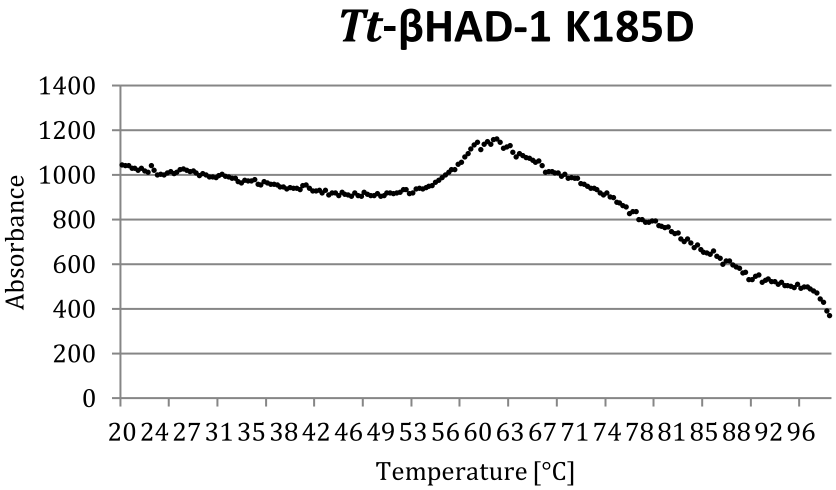

Figure S6: Thermofluor assay of *Tt*-βHAD-1 K185D

Figure S7: Thermofluor assay of *Tt*-βHAD-2 K187D

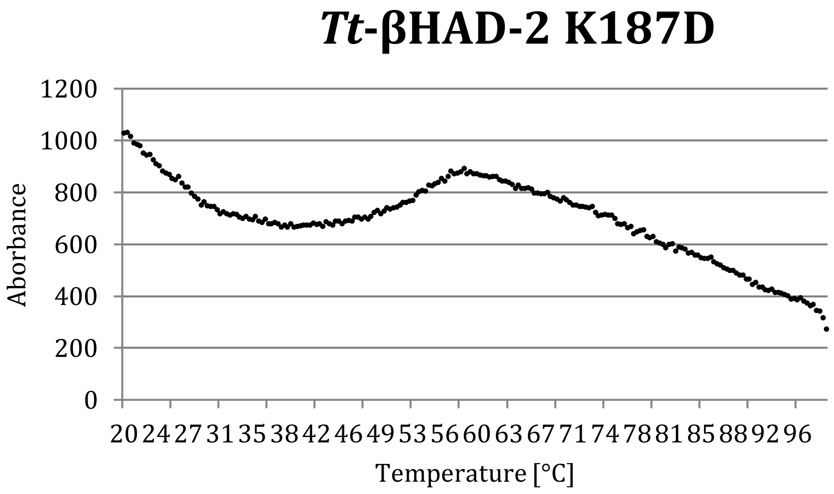

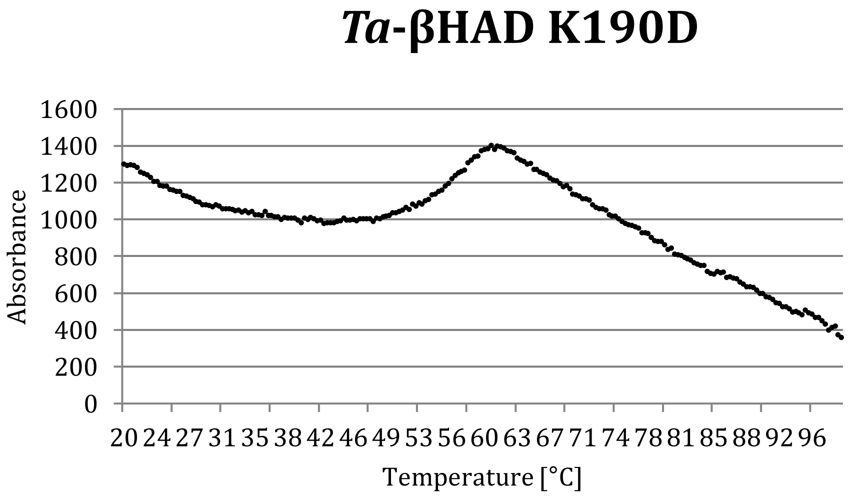

Figure S8: Thermofluor assay of *Ta*-βHAD K190D

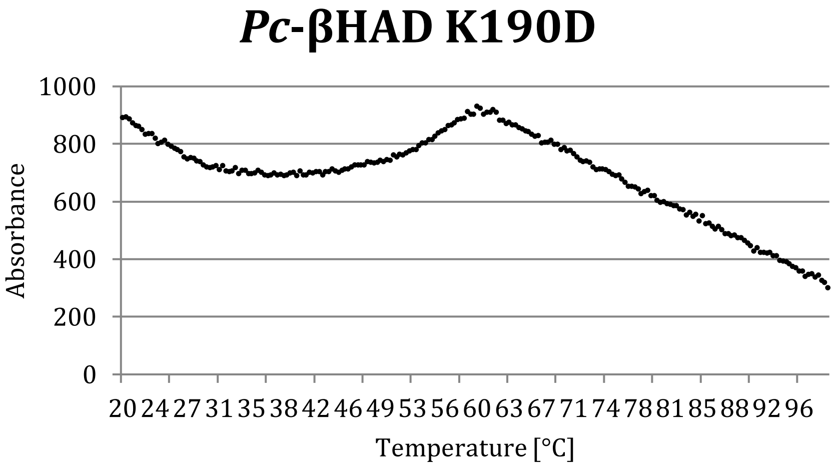

Figure S9: Thermofluor assay of *Pc*-βHAD K190D.

VII. Biotransformations

Biotransformations in co-solvents

Biotransformations in co-solvent were performed in 50 mM TRIS-HCl pH 8.0 and the respective co-solvent (5%, 10%, 25 %, 50% v/v) with purified enzyme (2 mg/mL) at 25 °C for 24 h. After 5 h were taken and analyzed for the biotransformations in methanol. The reaction consisted of 10 mM substrate, 2.5 mM NADPH, 25 mM glucose-6-phosphate and 5.0 U/mL glucose-6-phosphate-dehydrogenase. Negative controls were performed containing no βHAD, no substrate, or no NADPH. After 24 h, the reactions were stopped and analyzed as described elsewhere. The product formation was normalized to the product formation without any co-solvent.

Biotransformations

Biotransformations were performed in 1.5 mL reaction tubes (Sarstedt) with heat-purified enzyme and glucose-6-phosphate dehydrogenase cofactor regeneration system at 25°C and 600 rpm (Thermomixer comfort, Eppendorf) for all generated *Ta*-βHAD variants in triplicates the reactions were stopped at time points 0 h, 4 h, and 24h and analyzed as described elsewhere. The reaction mixture can be found in Table . As negative controls served buffer or empty vector.

Table S8: Reaction mix for biotransformations with *Ta*-βHAD variants

Components	150 µl reaction	Final concentration
TRIS-HCl-buffer (100 mM, pH 8.0)	45 µl	50 mM
500 mM Substrate**	1.5 µl	5 mM
250 mM NADPH*	1.5 µl	2.5 mM
1 M glucose-6-phosphate*	3.75 µl	25 mM
1000 U/mL glucose-6-phosphate dehydrogenase	0.75 µl	1 U/mL
250 mM $MgCl_2$*	1.5 µl	2.5 mM
Ta-βHAD variant#	60 µl	5 mg/mL
ddH_2O	36 µl	
*Dissolved in ddH_2O		
#Dissolved in TRIS-HCl-buffer (50 mM, pH 8.0 and 10% (*w/v*) L-sorbitol)		
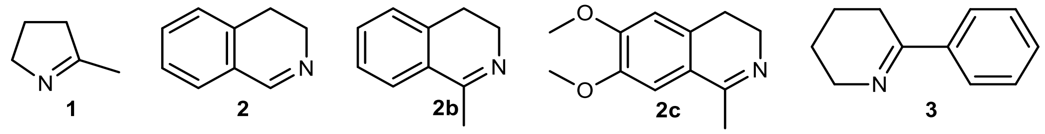 1, 2, 2b, 2c, 3		
**Substrate in MeOH (Substrate 1, 2, 2b, and 2c) or DMSO (Substrate 3)		

Sample preparation and extraction

Biotransformations with substrate **3** were analyzed with GC-FID. The biotransformations were stopped (at desired timepoint) by adding 50 µl 5M NaOH to each 150 µl biotransformation reaction. Then 400 µl methyl-*tert*-butyl ether (MTBE) was added as the extracting agent and 1 mM 3-methyl piperidine as an internal standard. The samples were immediately vortexed for 2 min and centrifuged for 5 min at 16000 g (Centrifuge 5415 R, Eppendorf) for phase separation. 200 µl Upper organic phase was transferred to GC vials with an inlet (WICOM).

For the analysis of the biotransformations via GC, the GC-2010 Plus gas chromatograph (Shimadzu) with a flame ionization detector (FID) was used. The applied conditions for substrates and corresponding products are shown in Table S7.

Biotransformations with substrate **2a** were analyzed with normal phase (NP) HPLC. The biotransformations were stopped (at the desired time point) by adding 50 µl 5M NaOH to each 150 µl biotransformation reaction. Then 400 µl cyclohexane-isopropanol mixture (ratio 70:30) was added as an extracting agent and 0.2 mM or 1 mM 1-acetonaphthone as an internal standard. The samples were immediately vortexed for 2 min and centrifuged for 5 min at 16000 g (Centrifuge 5415 R, Eppendorf) for phase separation. Then 200 µl upper organic phase was transferred to HPLC vials with an inlet (WICOM). For the analysis of the biotransformations via NP-HPLC an Agilent 1200 series HPLC system composed of degasser (G1379B, Agilent 1260 Infinity), quaternary pump (G1311A, Agilent 1200 series), autosampler (G1329A, Agilent 1200 series), thermostated column compartment (G1316A, Agilent 1200 series) and diode array detector (G1315D, Agilent 1200 series) was used. The applied conditions for substrates and the corresponding products are shown in Table SX.

The analysis of product formation was conducted by dividing the peak areas of the product by the IS area. The obtained quotient was then converted to a product concentration using a product standard curve.

Table S9: NP-HPLC setup for 3,4-dihydroisoquinoline and 1,2,3,4-tetrahydroisoquinoline

Substrate	3,4-dihydroisoquinoline
Product	1,2,3,4-tetrahydroisoquinoline
Internal standard	1-acetonaphthone
Column	CHIRALPAK® IC, 250 x 4,6 mm, 5 µm (Daicel)
Flow	1 ml/min
Solvent mixture	Cyclohexane:Isopropanol with 0.1% (*v/v*) diethylamine (ratio 80:20)
Temperature	25°C
Running time	10 min
Retention time	substrate: 7.1 min; product: 7.6 min; IS: 5.5 min
Wavelength	210 nm

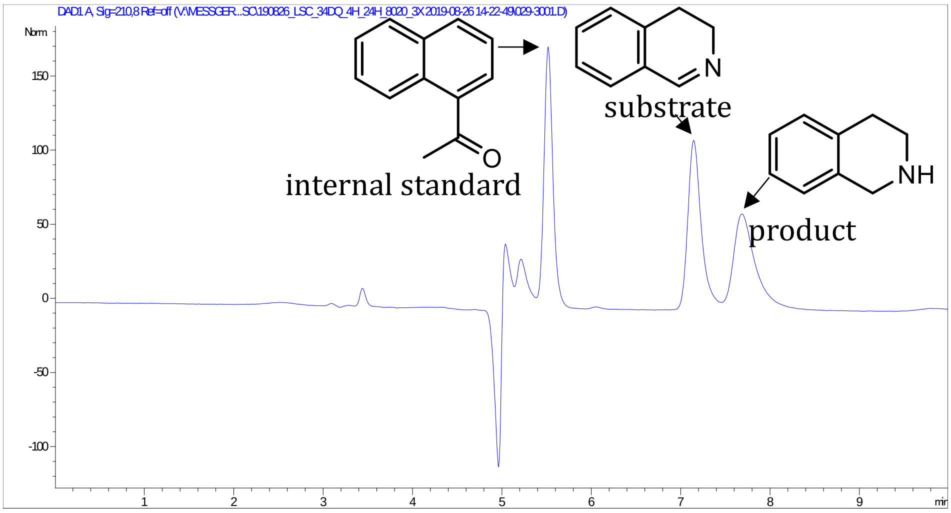

Figure S10: Exemplary HPLC chromatogram for quantification of product formation of the biotransformations of substrate **3** catalyzed by *Ta*-βHAD variant TA20.

Table S10: GC setup for 6-phenyl-2,3,4,5-tetrahydropyridine and 2-phenylpiperidine

Substrate 3	6-phenyl-2,3,4,5-tetrahydropyridine	
Product 3	2-phenylpiperidine	
Internal standard (IS)	3-methyl piperidine	
Column	ZB-5 (30m x 0,25 mm x 0,25 µm (Phenomenex))	
Temperature program		
Heating rate	Oven temperature	Holding time
	100°C	2 min
30°C/min	150°C	-
5°C/min	180°C	1 min
50°C/min	325°C	2 min
Carrier gas flow	H_2 30 cm/s	
Inlet temperature	250°C	
FID temperature	330°C	
Injection volume	1 µl	
Split	1:10	
Retention time	substrate: 7.8 min; product: 6.5 min; IS: 2.3 min	

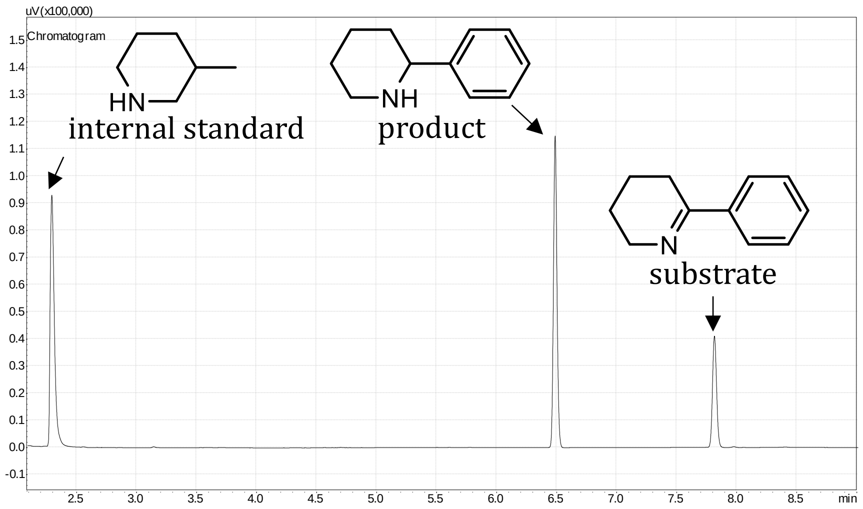

Figure S11: Exemplary GC-FID chromatogram for quantification of product formation of the biotransformations of substrate **3** catalyzed by *Ta*-βHAD variant TA20.

Table S11: Chiral NP-HPLC setup for 2-phenylpiperidine

Product	2-(*R*)-phenylpiperidine
Product	2-(*S*)-phenylpiperidine
Internal standard	1-acetonaphthone
Column	CHIRALPAK® IC, 250 x 4,6 mm, 5 µm (Daicel)
Flow	0.9 ml/min
Solvent mixture	Cyclohexane:Isopropanol with 0.1% (*v/v*) diethylamine (80:20)
Temperature	35°C
Running time	18 min
Retention time	*R*-product: 8.7 min; *S*-product: 9.1 min; IS:10.3 min
Wavelength	210 nm

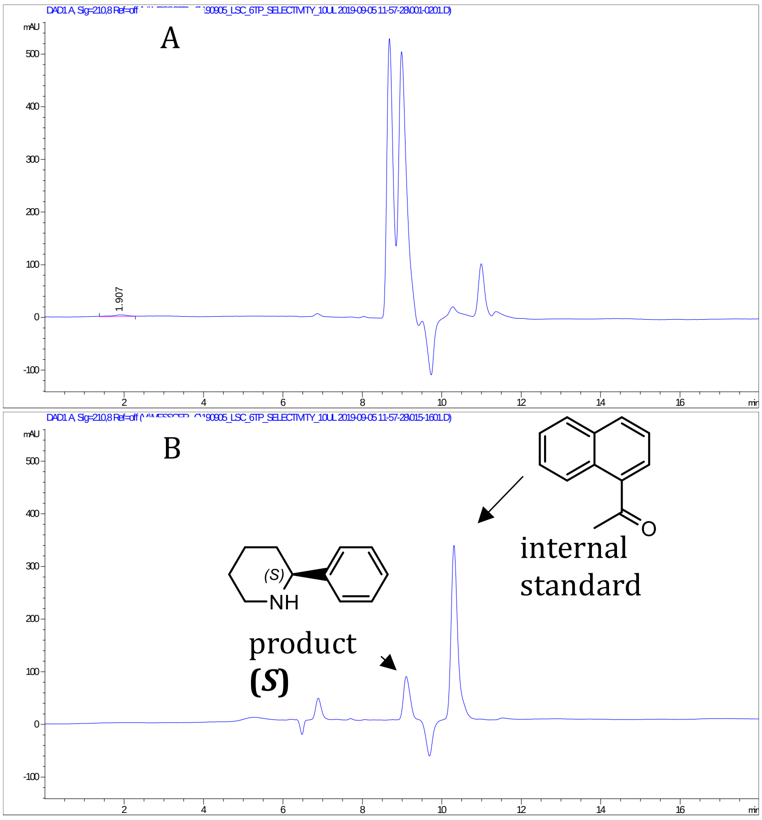

Figure S12: Exemplary Chiral NP-HPLC chromatograms to determine the *ee* value in the conversion of 3; **A**) racemic product reference; **B**) enzymatic reduction catalyzed by Ta-βHAD.

VIII. Molecular modeling

To visualize the residues selected for mutagenesis, a homology model of *Ta*-βHAD was generated. For this purpose, the Modeller[8,9] PlugIn PyMod2.0[10] was used with PyMOL 1.8.[11] For this purpose, the protein sequence (WP_012992122) of *Ta*-βHAD without His-tag and HRV 3C protease cutting site as input to perform a BLAST search[12] of the PDB.[13] The crystal structures of the in addition to that identified sequence homologs 3DOJ (30.0% sequence identity), 3PEF (33.1% sequence identity), and 3WS7 (35.7% sequence identity) were fetched, the chain's sequences were imported and aligned with *Ta*-βHAD sequence via MUSCLE.[14] Finally, a monomeric homology model was generated utilizing this cluster as a template (Figure S13), and its dimeric form was achieved via superposition of two monomeric copies to 3PEF_B and 3PEF_D, respectively (Figure S13). The NADP+ cofactor was obtained from 3WS7 and was adapted via PyMOL builder to provide a functional NADPH cofactor. The atom names were adapted manually. As all template sequences displayed a low sequence identity, energy minimization was performed to optimize the dimeric model. Parameters NADPH was calculated with antechamber.[15] Using the parameters listed in MOL2 and FRCMOD files, parameter XML files were generated. The pKa values of the side chains were calculated using PROPKA[16,17] provided by the PDB2PQR server (version 2.0.0).[18] A pH of 8 and the Parse forcefield were utilized. According to these results, all residues differing from the standard protonation state (D89, D114, K139, E179, K215, K258, E272) were protonated/deprotonated manually. The simulations were performed using OpenMM 7.4.1[19] utilizing NVIDIA CUDA GPU platform.[20] General Amber force field (GAFF) and Amber14 force field were used.[21,22] The cubic box with a padding of 1.5 nm was solvated with water (tip4p-Ew water model)[23], the protein charge was neutralized, and ionic strength of 0.1 M NaCl was applied, and a neutralized. Energy minimization was performed until 10 kJ/mole tolerance energy. A reference temperature of 300 K, a pH of 8, the Langevin integrator with a friction coefficient of 1/ps, and a step size of 2 fs were used.[24] In the resulting model the mutations of single variant TA1 (K189D; numbering includes His-tag and HRV 3C cutting site), triple variant TA6 (K189D/N192L/N193L; numbering includes His-tag and HRV 3C cutting site), quadruple variant TA16 (K189D/F250A/N192L/D258A; numbering includes His-tag and HRV 3C cutting site) and quintuple variant TA20 (K189D/F250A/N193L/N192L/D258S; numbering includes His-tag and HRV 3C cutting site) were introduced using the PyMOL mutagenesis tool.

A

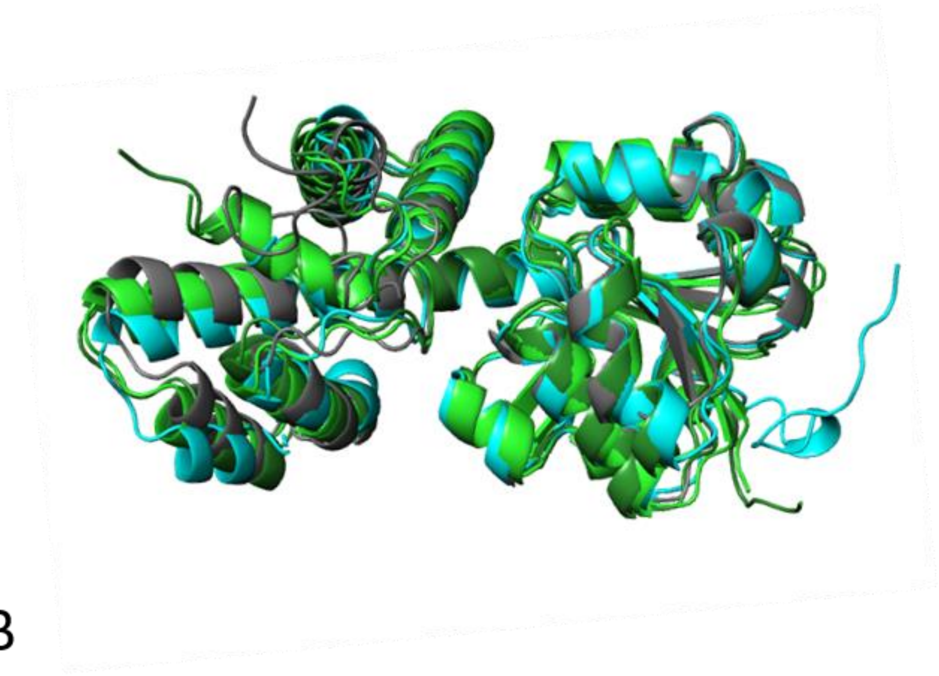

B

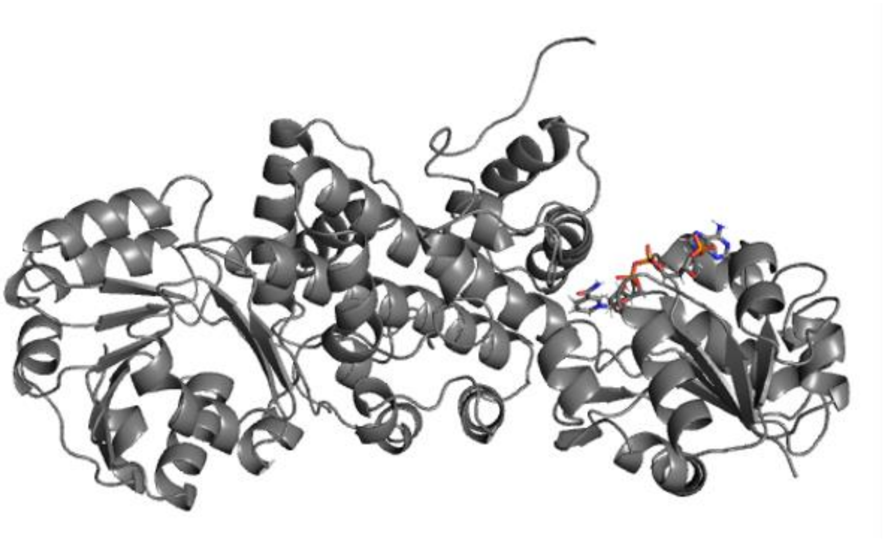

Figure S13: Homology model of *Ta*-βHAD (grey). (**A**) Monomeric form superimposed with the template structures 3DOJ (dark green), 3PEF (light green), and 3WS7 (cyan). (**B**) Dimeric structure with NADPH cofactor in monomer A.

IX. References

[1] S. Fademrecht, P. N. Scheller, B. M. Nestl, B. Hauer, J. Pleiss, *Proteins Struct. Funct. Bioinforma.* **2016**, *84*, 600–610.

[2] L. C. Reimer, A. Vetcininova, J. S. Carbasse, C. Söhngen, D. Gleim, C. Ebeling, J. Overmann, *Nucleic Acids Res.* **2019**, *47*, D631–D636.

[3] M. Lenz, S. Fademrecht, M. Sharma, J. Pleiss, G. Grogan, B. M. Nestl, *Protein Eng. Des. Sel.* **2018**, *31*, 109–120.

[4] Y. Zhang, Y. Zheng, L. Qin, S. Wang, G. W. Buchko, R. M. Garavito, *Biochimie* **2014**, *104*, 61–69.

[5] R. K. Njau, C. A. Herndon, J. W. Hawes, *Chem. Biol. Interact.* **2001**, *130*, 785–791.

[6] P. Rice, L. Longden, A. Bleasby, *Trends Genet.* **2000**, *16*, 276–277.

[7] J. Braman, C. Papworth, A. Greener, in *Vitr. Mutagen. Protoc.* (Ed.: M.K. Trower), Humana Press, Totowa, NJ, **1996**, pp. 31–44.

[8] A. Šali, *J. Mol. Biol.* **1993**, *234*, 779815.

[9] B. Webb, Š. Sali, *Curr. Protoc. Bioinformatics* **2014**, *1137*, 1–15.

[10] G. Janson, C. Zhang, M. G. Prado, A. Paiardini, *Bioinformatics* **2017**, *33*, 444–446.

[11] Schrödinger, LLC, *The PyMOL Molecular Graphics System, Version 1.8*, **2015**.

[12] S. F. Altschul, W. Gish, W. Miller, E. W. Myers, D. J. Lipman, *J. Mol. Biol.* **1990**, *215*, 403–410.

[13] H. M. Berman, T. Battistuz, T. N. Bhat, W. F. Bluhm, P. E. Bourne, K. Burkhardt, Z. Feng, G. L. Gilliland, L. Iype, S. Jain, P. Fagan, J. Marvin, D. Padilla, V. Ravichandran, B. Schneider, N. Thanki, H. Weissig, J. D. Westbrook, C. Zardecki, *Acta Crystallogr. Sect. D Biol. Crystallogr.* **2002**, *58*, 899–907.

[14] R. C. Edgar, *Nucleic Acids Res.* **2004**, *32*, 1792–1797.

[15] J. Wang, W. Wang, P. A Kollman, *J. Am. Chem. Soc* **2001**, *123*, 5221–5230.

[16] C. R. Søndergaard, M. H. M. Olsson, M. Rostkowski, J. H. Jensen, *J. Chem. Theory Comput.* **2011**, *7*, 2284–2295.

[17] M. H. M. Olsson, C. R. Søndergaard, M. Rostkowski, J. H. Jensen, *J. Chem. Theory Comput.* **2011**, *7*, 525–537.

[18] T. J. Dolinsky, J. E. Nielsen, J. A. McCammon, N. A. Baker, *Nucleic Acids Res.* **2004**, *32*, 665–667.

[19] P. Eastman, V. S. Pande, *Comput. Sci. Eng.* **2010**, *12*, 34–39.

[20] NVIDIA CUDA, *Comp. A J. Comp. Educ.* **2010,** *12*.

[21] J. Wang, R. M. Wolf, J. W. Caldwell, P. A. Kollman, D. A. Case, *J. Comput. Chem.* **2004**, *25*, 1157–1174.

[22] D. A. Case, V. Babin, J. T. Berryman, R. M. Betz, Q. Cai, D. S. Cerutti, T. E. Cheatham III, T. A. Darden, R. E. Duke, H. Gohlke, A. W. Goetz, S. Gusarov, N. Homeyer, P. Janowski, J. Kaus, I. Kolossváry, A. Kovalenko, T. S. Lee, S. LeGrand, T. Luchko, R. Luo, B. Madej, K. M. Merz, F. Paesani, D. R. Roe, A. Roitberg, C. Sagui, R. Salomen-Ferrer, G. Seabra, G. L. Simmerling, W. Smith, J. Swails, R. C. Walker, J. Wang, R. M. Wolf, X. Wu, P. A. Kollman, *AMBER 14,* **2014**.

[23] H. W. Horn, W. C. Swope, J. W. Pitera, J. D. Madura, T. J. Dick, G. L. Hura, T. Head-Gordon, *J. Chem. Phys.* **2004**, *120*, 9665–9678.

[24] G. Bussi, M. Parrinello, *Phys. Rev. E - Stat. Nonlinear, Soft Matter Phys.* **2007**, 75, 056707
